# A Novel Homozygous Intronic Variant in TNNT2 Associates With Feline Cardiomyopathy

**DOI:** 10.3389/fphys.2020.608473

**Published:** 2020-11-16

**Authors:** James W. McNamara, Maggie Schuckman, Richard C. Becker, Sakthivel Sadayappan

**Affiliations:** ^1^Division of Cardiovascular Health and Disease, Department of Internal Medicine, Heart, Lung and Vascular Institute, University of Cincinnati, Cincinnati, OH, United States; ^2^Department of Cardiology, MedVet Cincinnati, Fairfax, OH, United States

**Keywords:** Hypertrophic Cardiomyopathy, Maine Coon, MYBPC3, TNNT2, sarcomere

## Abstract

**Background:**

Hypertrophic cardiomyopathy (HCM) is a genetic disease of the heart and the most common cause of sudden cardiac death in the young. HCM is considered a disease of the sarcomere owing to the large number of mutations in genes encoding sarcomeric proteins. The riddle lies in discovering how these mutations lead to disease. As a result, treatments to prevent and/or treat HCM are limited to invasive surgical myectomies or ablations. The A31P variant of cardiac myosin binding protein-C, encoded by *MYBPC3*, was found to be more prevalent in a cohort of Maine Coon cats with HCM. However, other mutations in *MYBPC3* and *MYH7* have also been associated with HCM in cats of other breeds. In this study, we expand the spectrum of genes associated with HCM in cats.

**Results:**

Next Generation Whole Genome sequencing was performed using DNA isolated from peripheral blood of a Maine Coon with cardiomyopathy that tested negative for the *MYBPC3* A31P variant. Through risk stratification of variants, we identified a novel, homozygous intronic variant in cardiac troponin T (*TNNT2*). *In silico* analysis of the variant suggested that it may affect normal splicing of exon 3 of *TNNT2*. Both parents tested heterozygous for the mutation, but were unaffected by the disease. Echocardiography analyses revealed that the proband had shown early onset congestive heart failure, which is managed with a treatment regime including ACE and aldosterone inhibitors.

**Conclusion:**

In summary, we are the first to demonstrate the association between *TNNT2* mutations and HCM in felines, suggesting that this gene should be included in the testing panel of genes when performing genetic testing for HCM in cats.

## Introduction

Hypertrophic cardiomyopathy (HCM) is the most common form of genetic heart disease, affecting at least 1 in 500 humans (Ho, 2011). Defined by an unexplained abnormal thickening of the ventricular walls and possible outflow tract obstruction, no cure is currently available for HCM. HCM is primarily caused by mutations in genes that encode sarcomeric proteins. In particular, mutations in *MYH7* and *MYBPC3*, encoding beta-cardiac myosin heavy chain and cardiac myosin binding protein-C, respectively, are the most commonly mutated genes (Viswanathan et al., 2017), although other sarcomeric genes, including *TNNI3, TNNT2*, and *MYL2*, have also been implicated in humans (Brouwer et al., 2011). How mutations in these sarcomeric genes result in the development of HCM remains poorly understood.

In cats, cardiomyopathy is the main cause of cardiovascular disease, with HCM presenting as the most common form, suggesting that cats may be an excellent non-rodent animal model of HCM (Ferasin et al., 2003). A variant in *MYBPC3*, A31P, was previously linked to the development of HCM in Maine Coon cats (Meurs et al., 2005). This variant occurs within the C0 domain of cardiac myosin binding protein-C (cMyBP-C), and it is expected to cause disease *via* structural defects in this domain important for regulation of actomyosin regulation (Van Dijk et al., 2016). Another variant in *MYBPC3*, A74T, has also been described across multiple cat breeds. However, in-depth follow-up studies demonstrated that this polymorphism is unrelated to cardiomyopathy (Wess et al., 2010; Longeri et al., 2013). A third variant in *MYBPC3*, R820W, has been associated with HCM, most specifically in the Ragdoll (Meurs et al., 2007). More recently, the first variant in *MYH7*, E1883K, has been associated with HCM in a Domestic Shorthair cat, expanding the spectrum of genes associated with feline cardiomyopathy (Schipper et al., 2019). A full list of mutations associated with feline cardiomyopathy is shown in Table 1.

**TABLE 1:** The current list of known mutations associated with feline cardiomyopathy.

Gene	Mutation	Breed	References
MYBPC3	A31P	Maine Coon	Meurs et al. (2005)
MYBPC3	R820W	Ragdoll	Meurs et al. (2007)
MYH7	E1883K	Domestic Shorthair	Schipper et al. (2019)

In the present study, a male Maine Coon cat with cardiomyopathy and early congestive heart failure was studied. After initially screening negative for the aforementioned A31P mutation, we performed WGS to identify potential mutations and, thus, pinpoint the genetic cause of this cat’s cardiomyopathy through study of the diseased cat’s family tree. This study expands upon recent Next Generation Sequencing performed in cats (Genova et al., 2018; Ontiveros et al., 2018). As a result, we have identified a novel autosomal recessive mutation in the sarcomeric cardiac troponin T (*TNNT2)* gene that appeared to be associated with feline cardiomyopathy. This is the first known record of such mutation in felines, suggesting that *TNNT2* should be included as a candidate gene in the genetic screenings.

## Materials and Methods

### Phenotyping, Blood Collection and DNA Extraction

All procedures followed the protocol approved by the Institutional Animal Care and Use Committee of the University of Cincinnati and complied with the Guide for the Use and Care of Laboratory Animals published by the National Institutes of Health. The proband’s owner established contact to determine if the animal’s disease could be linked to a genetic mutation. To establish a full research study, a Material Transfer Agreement was initiated between the proband’s owner and the University of Cincinnati. Researchers had no contact with any animals beyond receipt of blood samples. All studies involving contact with animals were performed by board certified veterinarians. Diagnosis and treatment of the proband, regular checkups and echocardiography were performed by a board certified veterinary cardiologist using standard echocardiographic techniques and references from a population of healthy Maine Coon cats (Thomas et al., 1993; Drourr et al., 2005). Peripheral blood was collected by a certified veterinarian or veterinarian nurse at each animal’s local hospital following a routine check-up. Blood collected locally was transported back to the laboratory on wet ice. Blood that was collected at veterinary clinics farther from the lab was shipped on cold blocks overnight. Upon receipt at the laboratory, all blood samples were aliquoted into 200 μl samples and stored at −80°C until use. DNA was extracted from a single 200 μl aliquot using the Qiagen QIAamp DNA Mini and Blood DNA isolation kit, following the manufacturer’s protocol. Concentration and purity of DNA was analyzed by NanoDrop. For samples sent for WGS, DNA integrity and concentration were also determined using the Agilent 2100 Bioanalyzer and Qubit Fluorometer, respectively.

### Whole Genome Sequencing and Analysis

DNA extracted from the whole blood of the proband was submitted to Novogene Co., Ltd., for WGS. DNA quality and quantity were confirmed by Agarose gel electrophoresis and Qubit Fluorometer. A total amount of 1.5 μg high-quality genomic DNA was randomly sheared into short fragments of approximately 350 bp and used for library construction using the NEBNext^®^ DNA Library Prep Kit. Briefly, following end repairing, dA-tailing, and ligation with NEBNext adapter, the fragments were PCR-enriched by P5 and indexed P7 oligos. The concentration of the DNA library was quantified using the Qubit 2.0 fluorometer and diluted to 1 ng/μl. Following dilution, the insert size of the library was assessed with the Agilent 2100 bioanalyzer, and quantitative real-time PCR (qPCR) was performed to detect the effective concentration of the prepared library. Following enrichment and indexing, pair-end sequencing of the qualified library was performed on an Illumina HiSeq X Ten with a read length of PE150bp at each end.

### Bioinformatics Analysis

The original sequencing data acquired by high-throughput sequencing platforms (e.g., Illumina HiSeq^TM^/NovaSeq^TM^) and recorded in image files were first transformed to sequence reads by base calling with the CASAVA software. Information on sequences and corresponding sequencing quality was stored in a FASTQ file. Following quality control of raw sequencing data for clean data filtration, each clean read was mapped to the reference genome (Felis_Catus_9.0, Ensembl release 93) using the BWA software (Li and Durbin, 2009), and the mapping rate and coverage were counted according to the alignment results. Duplicates were removed by SAMTOOLS (Li et al., 2009). Single nucleotide polymorphism (SNP) and InDel variants were detected using the GATK software (Depristo et al., 2011). These SNPs were annotated using ANNOVAR as previously described (Wang et al., 2010).

### Sanger Sequencing

To test, or confirm, the presence of a genetic variant in parents of the proband, PCR was performed using a high-fidelity *Taq* polymerase to specifically amplify the sequence around the variant. The PCR product was purified and sent for Sanger sequencing using Cincinnati Children’s Hospital DNA Sequencing and Genotyping Core Facility.

## Results

**FIGURE 1:**
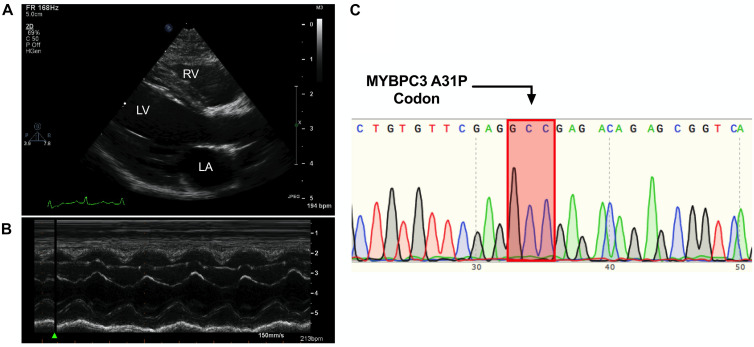
Cardiomyopathy in the absence of the *MYBPC3 A31P* variant. **(A,B)**, Representative echocardiography and outline of cardiovascular disease risk stratification of genetic variants. Representative parasternal long axis B-Mode **(A)** and M-Mode **(B)** images of proband, with left ventricle (LV), left atrium (LA) and right ventricle (RV) labeled. **(C)**, Chromatogram of *MYBPC3* A31P demonstrated that the proband was negative for this variant.

The proband was a privately-owned male pure-bred Maine Coon. At 8 months of age, he presented with left ventricular, right atrial, and borderline left atrial dilatation and borderline septal hypertrophy, with preserved-to-elevated systolic function (Figures 1A,B and Table 2), when compared to reference echocardiography values for the Maine Coon (Drourr et al., 2005). Diastolic function could not be quantified as a result of fusion of E and A waves. Progressive enlargement of all four chambers of the heart was noted at 14 months of age, while borderline septal hypertrophy and preserved systolic function was still noted. This was diagnosed as a primary unclassified cardiomyopathy with possible early congestive heart failure. Furosemide, Benazepril, Spironolactone, and Clopidogrel treatments were then initiated by the veterinarian. This combination appeared to stabilize the progression of the heart disease at 18 months (Table 2).

**TABLE 2:** Echocardiography parameters from serial measurements performed by a board-certified veterinary cardiologist.

Parameter	Proband				References
Age (month)	10	14	18	24	>12
IVSd (mm)	6	5.9	5.88	5.94	3.9–4.1
LVIDd (mm)	19.3	19.9	21.8	21.7	18.1–18.9
LVPWd (mm)	5.1	5.5	5.27	5.55	4.2–4.4
IVSs (mm)	9.6	9.2	N/A	N/A	7.2–7.8
LVIDs (mm)	9	9.6	7.79	8.11	8.5–9.3
LVPWs (mm)	9.6	9.4	N/A	N/A	7.8–8.2
%FS	60.6	59.5	64.2	62.6	50.35–53.35
LA (mm)	17.6	19.8	18.9	19.7	13.4–14.0
LA/Ao	1.33	1.58	1.53	1.44	1.20–1.26
Treatments		Furosemide, Benazepril, Clopidogrel	Spironolactone, Furosemide, Benazepril, Clopidogrel	Spironolactone, Furosemide, Benazepril, Clopidogrel	

*Significant dilatation of all cardiac chambers was noted with normal to elevated systolic function. Treatments administered are listed. IVS, interventricular septal thickness; LVID, left ventricular internal diameter; LVPW, left ventricular posterior wall thickness; FS, fractional shortening; LA, left atrial dimension; LA/Ao Left atrium-to-aortic root ratio; d, diastole; s, systole. N/A represents data not available.*

DNA was isolated from whole peripheral blood from the proband. PCR was performed to produce a 251 amplicon around the A31P variant in *MYBPC3* previously associated with HCM in Maine Coon cats (primer sequences in Table 3). Sanger sequencing of the PCR product showed no mutation at codon 31, indicating that the proband did not carry the pathogenic A31P variant in *MYBPC3* (Figure 1C).

**TABLE 3:** List of primer sequences used in this study to test for the presence of variants in *MYBPC3* and *TNNT2 genes*.

Primer name	Primer sequence	Amplicon size (bp)
Cat_A31P_F	AGTCTCAGCCTTCAGCAAGAAGCC	252 bp
Cat_A31P_R	GGTCAAACTTGACCTTGGAGGAGCC	
Cat_TNNT2_F	TGAGTGGATGTGGCTGTGTT	287 bp

**FIGURE 2:**
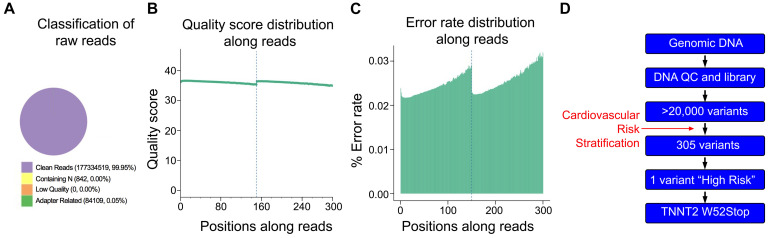
Quality control of Next Generation Sequencing results. **(A)** From 177,419,470 sequencing reads, 99.95% were clean reads, while just 0.05% contained adapter-related contaminations. **(B)** Quantification of sequencing quality distribution along reads demonstrated high-quality scores along entire reads with 94.19% of base reads having a Phred score greater than 30. **(C)** Error rate distribution along reads demonstrated high-fidelity reads with an overall error rate of 0.03%. **(D)** Cardiomyopathy gene stratification strategy using 174 genes from the cardio panel gene list.

As the proband was negative for the A31P mutation in *MYBPC3*, we asked if any other variants could be traced to the proband’s cardiomyopathy. Accordingly, DNA was freshly isolated for WGS to identify potential variants present in the proband. High-quality DNA with an approximate size of 22944bp was sent for library preparation and sequencing by Novogene. Sequences were aligned against the Felis Catus reference genome (Ensemble release 93). The sequencing resulted in 177419470 raw reads, with an average depth of 17.59 reads per base, an error rate of 0.03, and 94.19% of base reads with a Phred score greater than 30 (Figures 2A–C). The detected GC content was 43.06% compared to the reference genome of 41.73%. Together, these data demonstrated sufficiently high quality of sequencing data.

**FIGURE 3:**
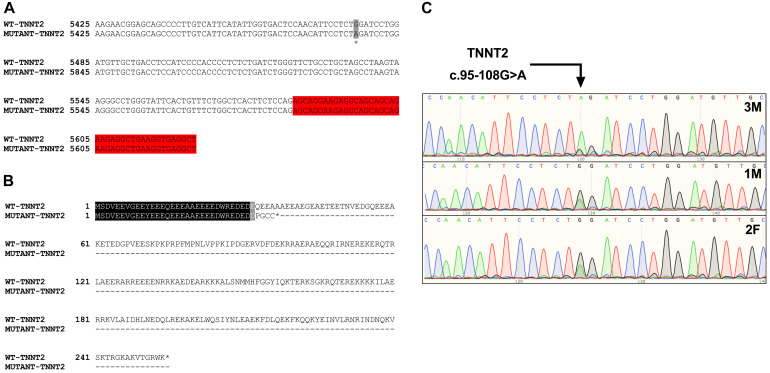
Analysis of the *TNNT2* c.95-108G > A variant. **(A)** Gene sequence alignment of wild-type and mutant *TNNT2*. The variant is highlighted in grey, and the downstream exon is highlighted in red. **(B)** Protein sequence alignment of the wild-type TNNT2 protein and the predicted protein resulting from the *TNNT2* c.95-108G > A variant. **(C)** Sanger sequencing chromatograms demonstrating homozygosity for the *TNNT2* c.95-108G > A variant in the proband and heterozygosity in both parents.

Initial analysis reported more than 20,000 genetic variants identified from the proband, compared to the reference genome. We employed a cardiovascular disease risk stratification step in order to enrich for variants associated with cardiovascular disease. This stratification was with 174 genes covered by the Illumina TruSight Cardio Panel (Pua et al., 2016). This approach reduced the number of variants to 305 (Figure 2D). Of these 305 potential variants, only one had been annotated as having “high risk” impact. This particular variant was a single base pair substitution (G to A) within intron 3, corresponding to c.95-108G > A of ENSFCAG00000004613. This variant resulted in the potential inclusion of a *de novo* splice acceptor site (Figure 3A). We then used *in silico* analysis to determine the likelihood that this intronic variant indeed acts as a novel splice acceptor site (Wang and Marín, 2006). This *in silico* method correctly identified greater than 96% of canonical splice donors and acceptors within *TNNT2* (Table 4). Of note, *in silico* analysis of the c.95-108G > A variant in the proband supported the hypothesis that this variant alters *TNNT2* splicing. The consequence of this aberrant splicing was investigated at the protein level, which revealed that the variant could cause the loss of 223 carboxyl terminal amino acids and incorporation of only five novel amino acids, rendering the protein non-functional (Figure 3B).

**TABLE 4:** *In silico* prediction of exon splicing sites of cat *TNNT2* (ENSFCAG00000004613) gene.

*In silico* Exon splicing prediction			
**Position**	**Predicted Splice**	**Score**	**Confidence**
Exon 1 Donor	Constitutive donor	11.931	0.332
Exon 2 Acceptor	Alt. isoform/cryptic acceptor	5.476	0.495
Exon 2 Donor	Constitutive donor	11.4	0.405
Exon 3 **de novo** Acceptor	Constitutive acceptor	4.688	0.396
Exon 3 Acceptor	Alt. isoform/cryptic acceptor	6.649	0.509
Exon 3 Donor	Constitutive donor	13.878	0.871
Exon 4 Acceptor	Constitutive acceptor	5.828	0.568
Exon 4 Donor	Constitutive donor	15.502	0.523
Exon 5 Acceptor	Constitutive acceptor	12.683	0.888
Exon 5 Donor	Constitutive donor	15.343	0.801
Exon 6 Acceptor	Constitutive acceptor	10.572	0.727
Exon 6 Donor	Alt. isoform/cryptic donor	10.982	0.805
Exon 7 Acceptor	Constitutive acceptor	8.308	0.85
Exon 7 Donor	Alt. isoform/cryptic donor	8.771	0.782
Exon 8 Acceptor	Constitutive acceptor	10.214	0.924
Exon 8 Donor	Constitutive donor	13.02	0.872
Exon 9 Acceptor	Not Predicted	N/A	N/A
Exon 9 Donor	Alt. isoform/cryptic donor	11.345	0.347
Exon 10 Acceptor	Constitutive acceptor	8.201	0.438
Exon 10 Donor	Constitutive donor	11.746	0.511
Exon 11 Acceptor	Constitutive acceptor	8.577	0.643
Exon 11 Donor	Alt. isoform/cryptic donor	10.946	0.235
Exon 12 Acceptor	Constitutive acceptor	8.91	0.812
Exon 12 Donor	Constitutive donor	8.271	0.728
Exon 13 Acceptor	Constitutive acceptor	11.353	0.443
Exon 13 Donor	Constitutive donor	12.265	0.581
Exon 14 Acceptor	Constitutive acceptor	10.067	0.841

**FIGURE 4:**
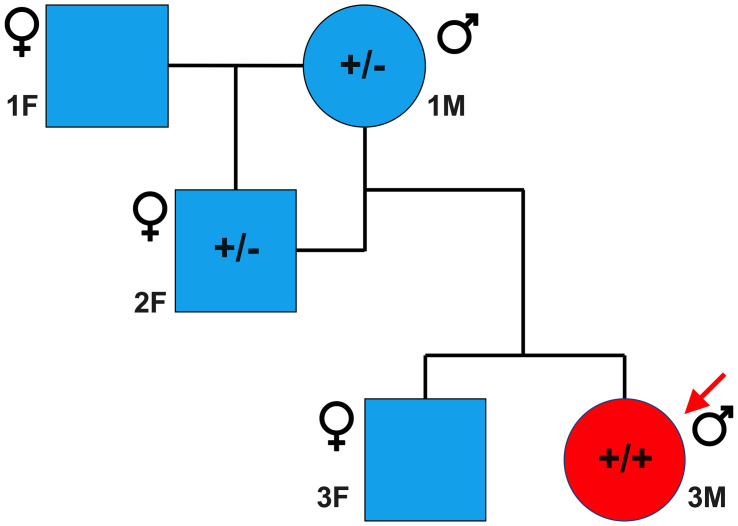
Family pedigree of affected proband. Circles and squares represent males and females, respectively, while blue and red represent unaffected and affected individuals, respectively. A male Maine Coon, heterozygous for the *TNNT2* c.95-108G > A variant, was mated to a female Maine Coon, which resulted in one female offspring. This female offspring, also heterozygous, was then bred with the original male, producing two offspring. The male offspring was the cardiomyopathic proband in this study and was homozygous for the *TNNT2* c.95-108G > A variant. * indicates that samples from these cats were not available at the time of the study.

Sanger sequencing was performed to validate the WGS annotation of the c.95-108G > A variant in the proband (primer sequence in Table 3). Strikingly, this sequencing revealed homozygosity of the c.95-108G > A variant in the proband, further suggesting its disease association (Figure 3C). Following this finding, blood samples from both parents of the proband were obtained. Echocardiography did not suggest any cardiac pathology in either of these parents (data not shown). DNA was extracted and PCR performed for Sanger sequencing of the *TNNT2* variant. Strikingly, both parents harbored a single copy of the c.95-108G > A variant (Figure 3C). This finding led to investigations into the pedigree of the proband. It was found that the proband was born from consanguineous breeding (Figure 4). Taken together, these results implicate a novel intronic mutation which results in the truncation of the sarcomeric protein, cardiac troponin T, as the cause of cardiomyopathy in this Maine Coon cat.

## Discussion

In this study, we identified a novel homozygous intronic variant in *TNNT2* which was associated with a case of feline cardiomyopathy and early heart failure. Mutations in *MYBPC3* and *MYH7* have previously been described. However, to the best of our knowledge, this is the first report of a thin filament mutation associated with feline cardiomyopathy (Meurs et al., 2005; Schipper et al., 2019). Mutations in *TNNT2* have been strongly implicated in the development of HCM and dilated cardiomyopathy (Watkins et al., 1995; Pasquale et al., 2012). In humans, these *TNNT2* mutations are described as phenotypically mild compared to *MYH7* mutations, but with a higher incidence of sudden cardiac death (Watkins et al., 1995). Importantly, mutations in intronic splice sites have been identified as causative of cardiomyopathy in humans (Thierfelder et al., 1994). Taken together, these data support our finding that the *TNNT2* intronic mutation we describe is the most likely cause of this case of feline cardiomyopathy.

The *TNNT2* gene encodes the cardiac-specific sarcomeric protein cardiac troponin-T (cTnT) (Wei and Jin, 2016). Cardiac troponin-C and -I (cTnC and cTnI, respectively), together with cTnT, make up the troponin complex of the heart (Gomes et al., 2002). The troponin complex is associated with the thin filaments, and cTnT is understood to play an important role in anchoring the complex to both actin and tropomyosin (Gomes et al., 2002). When calcium is released from the sarcoplasmic reticulum, it binds to and induces a structural change in cTnC (Vinogradova et al., 2005), ultimately leading to the movement of tropomyosin, allowing the formation of cross-bridges (Boussouf et al., 2007). cTnT has been demonstrated to regulate the calcium sensitivity of actomyosin ATPase and force (Gomes et al., 2005). Thus, cTnT has a central role in regulating contraction and relaxation of the heart.

Homozygous knockout of the *TNNT2* gene in mice is embryonically lethal (Ahmad et al., 2008; Nishii et al., 2008). As such, it is unlikely that the *de novo* splice acceptor site in the mutant *TNNT2* is fully penetrant. Rather, it is more likely that the aberrant splicing of *TNNT2* is a limited event, resulting in haploinsufficiency of the cTnT protein. This variant results in the significant truncation of the cTnT protein. It is well known that both the middle carboxyl-terminal regions of cTnT are required for its interaction with tropomyosin, cTnI, and cTnC (Wei and Jin, 2016). Therefore, the loss of these regions would likely prevent the incorporation of the truncated protein into the sarcomere. The resultant reduction in cTnT levels would result in an abnormal stoichiometry of thin filament proteins in the sarcomere, which would be sufficient to cause cardiomyopathy. Indeed, haploinsufficiency of cTnT has previously been associated with cardiomyopathy (Bollen et al., 2017). Furthermore, the incorporation of less than 5% of C-terminally truncated cTnT is sufficient to cause cardiomyopathy in mice (Tardiff et al., 1998). Since the proband’s symptoms are currently managed *via* treatment, consisting of Furosemide, Spironolactone, Benazepril and Clopidogrel, we cannot confirm the exact level of haploinsufficiency. Unfortunately, DNA from the proband’s littermate was not available, but we predict it would be either negative or heterozygous for the *TNNT2* c.95-108G > A variant. While no variants were identified in *MYH6* or *MYH7*, two single nucleotide variants were also identified in *MYBPC3* that were classified as having moderate risk. These variants were a proline substituted to a leucine at position 922 (P922L) and an alanine for a threonine at amino acid 1037 (A1037T), occurring within domains C7 and C8 of cMyBP-C, respectively. However, further investigation of these variants revealed that these amino acids are not evolutionarily conserved, and, furthermore, the substituted amino acids matched those of either human or mouse, effectively ruling out these *MYBPC3* variants as disease-causing.

## Conclusion

We have identified a novel, homozygous mutation in the sarcomeric gene *TNNT2*, which is associated with cardiomyopathy in a Maine Coon cat. The homozygosity of this mutation resulted from inbreeding. This study is the first to describe a mutation in *TNNT2* that is associated with feline cardiomyopathy, suggesting that this gene should be included in routine genetic testing in felines. Additionally, breeders should be mindful of the dangers associated with close generational consanguineous breeding.

## Data Availability Statement

The sequencing data has been deposited into the BioProject database (accession: PRJNA671288).

## Ethics Statement

The animal study was reviewed and approved by Ref: AM07-19-10-03-01 Institutional Animal Care and Use Committee OLAW Assurance D16-00190, AAALAC 00278 University of Cincinnati PO Box 210572, Cincinnati, OH 45267-0572. Written informed consent was obtained from the owner for the participation of their animals in this study.

## Author Contributions

JM and SS designed the research and wrote the manuscript. JM and MS performed the research. JM, MS, and SS analyzed data. MS and RB edited the manuscript. RB provided project oversight for clinical data. All authors read and approved the final manuscript.

## Conflict of Interest

SS provided consulting and collaborative research studies to the Leducq Foundation, Red Saree Inc., Greater Cincinnati Tamil Sangam, MyoKardia, Merck and Amgen, but such work is unrelated to the content of this manuscript. No other disclosures are reported. RB serves on scientific advisory boards for Janssen and Basking Biosciences and DSMB Committees for Ionis Pharmaceuticals, Akcea Therapeutics and Novartis. The remaining authors declare that the research was conducted in the absence of any commercial or financial relationships that could be construed as a potential conflict of interest.
